# Potential of RNA-binding protein human antigen R as a driver of osteogenic differentiation in osteoporosis

**DOI:** 10.1186/s13018-022-03073-w

**Published:** 2022-04-12

**Authors:** Zelin Liu, Baitao Li, Hai Hu, Xiaodong Li, Xiaofeng Zhang

**Affiliations:** 1 Department of Orthopedics and Traumatology, Second Affiliated Hospital, Heilongjiang University of Chinese Medicine, Harbin, 150000 China; 2 Department of Acupuncture, First Affiliated Hospital, Heilongjiang University of Chinese Medicine, Harbin, 150000 China; 3 Heilongjiang University of Chinese Medicine, No. 24, Heping Road, Xiangfang District, Harbin, 150000 Heilongjiang Province China

**Keywords:** HuR, LRP6, Osteoporosis, Osteogenic differentiation, Apoptosis

## Abstract

**Background:**

Emerging evidence has correlated the human antigen R (HuR) with the low-density lipoprotein receptor-related protein 6 (LRP6) gene, an important therapeutic target for osteoporosis. Herein, we sought to probe the regulatory role of HuR in the LRP6 gene and their interaction in the progression of osteoporosis.

**Methods:**

HuR and downstream potential target genes were predicted by bioinformatics analysis to identify their potential functions in bone metabolism following osteoporosis. The effect of HuR on the osteoblastic differentiation and viability and apoptosis of mouse embryo osteoblast precursor cells (MC3T3-E1) was evaluated after artificial modulation of HuR expression.

**Results:**

Bone phenotypes were observed in ovariectomized mice in response to adenovirus-mediated HuR overexpression. Poor expression of HuR was identified in the bone tissues of ovariectomized mice. Silencing of HuR inhibited the osteoblastic differentiation of MC3T3-E1 cells, as evidenced by decreased expression of Runx2 and Osterix along with reduced ALP activity. Mechanistically, HuR stabilized LRP6 mRNA and promoted its translation by binding to the 3'UTR of LRP6 mRNA, leading to activation of the downstream Wnt pathway. By this mechanism, osteoblastic differentiation of MC3T3-E1 cells was induced. In ovariectomized mice, overexpression of HuR alleviated osteoporosis-related phenotypes.

**Conclusion:**

Overall, these data together support the promoting role of HuR in the osteoblastic differentiation, highlighting a potential novel strategy for osteoporosis treatment.

**Supplementary Information:**

The online version contains supplementary material available at 10.1186/s13018-022-03073-w.

## Background

Osteoporosis is a progressive bone disease that is characterized by diminished bone density and microstructural damage, which increases the risk of fractures [1]. Postmenopausal osteoporosis is, in particular, an alarming health problem worldwide, afflicting up to 50% women after the age of 55 years [2]. The bone-forming cell, osteoblast, is of critical significance for the growing and maintaining of skeletons, and the importance of osteoblasts leads to growing studies with new model systems to fully understand osteoblasts impact metabolic bone diseases [3]. In addition, accumulating evidence proposed pivotal molecules orchestrating the coordination of osteoblast behaviors in the process of osteogenic differentiation and bone remodeling [4]. Improved understanding of osteogenic differentiation at molecular and cellular levels could generate new knowledge on pathophysiologic conditions of bones.

Human antigen R (HuR) is an RNA-binding protein that regulates a variety of post-transcriptional processes, including RNA stability [5, 6]. HuR participates in an array of cellular events, including proliferation, differentiation, senescence, apoptosis, and immune responses owing to its regulation of target transcripts [7]. Targeting HuR can serve as a potent therapeutic target to treat several diseases, especially cancer [8]. In addition, deficiency of HuR triggers a reduction in the expression of LRP6 in the mucosal tissues [9].

Of interest, LRP6 has been considered as an important therapeutic target for multiple diseases, particularly for osteoporosis [10]. Meanwhile, reduction of the LRP6 expression disrupts the osteogenic differentiation in pre-osteoblasts MC-3T3 [11]. A recent study has demonstrated LRP6 to be a canonical Wnt pathway co-receptor and that LRP6 can activate the Wnt pathway in Caco-2 cells [12]. The Wnt pathway has been report to play a pivotal role in maintaining bone mass [13]. Wnt/β-catenin pathway participates in a variety of physiological processes by modulating downstream target genes, including bone differentiation [14]. GSK3β is an important regulator of cell differentiation and apoptosis in the Wnt/β-catenin pathway and functions as a regulator of the balance between bone cells and osteoclasts [15]. Activation of the Wnt pathway diminishes the activity GSK3β, allowing β-catenin and Gli transcriptional factor to enter the nucleus and thereby augmenting the expression of downstream genes [14].

LiCl is a class of drugs and can result in increased bone mass and decreased bone turnover [16]. Therefore, LiCl may be used as a drug candidate for osteoporosis, but its therapeutic mechanism remains unclear. Further, a previous study has established that LiCl, as an inhibitor of GSK3β, can activate the Wnt pathway by repressing GSK3β, promote the bone formation of bone marrow-derived mesenchymal stem cells (BMSCs), and attenuate adipogenesis [14]. Furthermore, the Wnt/β-catenin pathway has been involved in osteoblast proliferation [17] and osseointegration [18].

Based on previous reports, this study aims to investigate the role of HuR based on its correlation with LRP6 and the Wnt pathway in bone formation in ovariectomized (OVX)-induced mouse models of osteoporosis, so as to deepen our understanding of the underlying mechanism of osteoporosis and provide novel therapeutic targets.

## Materials and methods

### Ethics statement

Animals were treated humanely in compliance with the recommendations in the *Guide for the Care and Use of Laboratory Animals* issued by the US National Institutes of Health. Animal experiments were approved by the Laboratory Animals Use and Management Committee of Heilongjiang University of Chinese Medicine (NO.2020061103).

### In silico* prediction*

Putative target mRNAs of the mouse RNA-binding protein HuR were retrieved from the CLIPdb2 database using Piranha and CIMS tools. The obtained mRNAs were then subjected to GO and KEGG enrichment analysis. The RPISeq tool was used to predict the interaction between mouse RNA-binding protein HuR and the downstream target genes.

### Adenovirus-mediated gene overexpression

The coding DNA sequence of HuR was retrieved from the NCBI database to synthesize the sequence of target genes. The synthesized target genes and shuttle plasmids were constructed, after which the shuttle plasmids and adenoviral backbone plasmids were amplified and co-transduced into 293A cells for recombination and adenovirus preliminary packaging. Approximately 15 days later, when the adenovirus-specific cytopathic effect (CPE) occurred in the packaged cells, the cytopathic plaques were collected and transferred into fresh 293A cells for a small amount of virus amplification. When the CPE occurred again, the cell supernatant was collected, and freeze–thaw was repeated three times to collect virus, which was designated as passage 1 (P1). Subsequently, 293A cells were transduced with the P1 adenovirus, with three consecutive passages. The P4 adenovirus was then amplified at a large scale and upon CPE occurrence, adenovirus was collected and concentrated in large proportion in vitro, with the target gene-carried adenovirus with a titer of 10^10^ PFU/mL obtained. Moreover, for animal experiments, adenovirus purification was performed with CsCl density gradient centrifugation to obtain the target gene-carried adenovirus at a titer of 10^11^ PFU/mL.

### Establishment of OVX-induced mouse models of osteoporosis

A total of 60 sixteen-week-old C57BL/6 mice (Vital River Laboratories, Beijing, China), weighing 20–22 g, were housed under specific pathogen-free conditions (22 ± 1 °C, 55 ± 10% humidity, 12-h light/dark cycle) with free access to food and water.

A week before model establishment, the bone density of mice was determined with X-ray bone densitometer. The mice were subjected to ovariectomy at the age of 3 months. Mice were sham-operated (n = 12) or OVX-induced for osteoporosis modeling (n = 12). Another three groups of mice (n = 12), including sham-operated mice and OVX-exposed mice with/without injection of HuR overexpressing BMSCs, were prepared for the subsequent micro-CT detection, tissue section, and mRNA detection.

OVX to induce osteoporosis was performed as previously documented [19]. Briefly, mice were anesthetized using 1.5% isoflurane (0.5–1.0 L/min). The hair in the dorsal mid-lumbar area of mice was shaved, and a 20-mm midline dorsal skin incision was subsequently made, after which a pair of ovaries were excised and the wound was sutured. In contrast, sham-operated mice underwent the same procedure except for the removal of ovaries.

One month after the establishment of OVX models, the mice were injected with BMSCs overexpressing HuR or the corresponding control vector NC. BMSCs were pre-treated as follows: BMSCs were transduced with adenovirus carrying oe-HuR or vector NC with an effective titer of 10^11^ PFU/mL. The overexpression efficiency of HuR was detected after 48 h. Next, 1 × 10^5^ BMSCs transduced with adenovirus carrying oe-HuR or vector NC were injected into the femur of the OVX-induced mice. The mice were euthanized 8 weeks later for subsequent experiments [20, 21].

### Hematoxylin and eosin (H&E) staining

Paraffin-embedded mouse femoral tissues were cut into tissue blocks and then sectioned at a thickness of 6–10 μm. The sections were fixed in 2% glutaraldehyde at 4 °C for 24 h, decalcified with 10% ethylenediaminetetraacetic acid at room temperature for 2 weeks, and then fixed with 1% osmium tetraoxide at 4 °C for 12 h. Thereafter, the sections were treated with 3% methanol in H_2_O_2_ for 20 min. The sections were stained with hematoxylin for 5–10 min, and with 0.5–1.0% eosin for 1 min, and counterstained with hematoxylin for 1 min. Sections were blued in 1% ammonia water, dehydrated, permeabilized with xylene and sealed with neutral resin. Finally, the sections were observed and photographed under a microscope in 5 randomly selected high-power fields from each section, with 100 cells counted in each field.

### Enzyme-linked immunosorbent assay (ELISA)

The levels of osteoblast- and the Wnt pathway-related factors in the serum of sham-operated mice, untreated OVX mice, or OVX mice treated with oe-HuR were determined by ELISA kits according to manufacturer’s protocols.

### Isolation and culture of primary BMSCs

Newborn mice were euthanized by cervical dislocation under anesthesia and soaked in 75% ethanol for 5 min. Under aseptic conditions, the bilateral femur was removed from the mice, after which the attached tissue was removed, and both ends were cut to expose the bone marrow cavity. Low-glucose Dulbecco's modified Eagle's medium (DMEM) was pipetted using a 1-mL syringe to rinse the femur repeatedly until it became white. The collected cell suspension was filtered through a 200-mesh sieve to prepare a single cell suspension. After counting, 1 × 10^5^ cells/mL were seeded into a culture flask containing low-glucose DMEM containing 20% FBS and cultured in a 5% CO_2_ incubator at 37 °C.

Half of the medium was renewed after 5 days, and the entire medium was renewed every 3–4 days. Upon reaching 80–90% confluence, the cells were detached with 0.25% trypsin for 30 s and observed under an inverted phase contrast microscope. Next, trypsin was discarded and FBS was added to terminate the digestion. The cells were added with low-glucose DMEM containing 20% FBS, and gently dissociated into single cell suspension with an elbow straw. The cells were resuspended in a culture flask, designated as passage 0 (P0), and cultured with 5% CO_2_ at 37 °C. After 48 h, the medium was changed. When reaching 80–90% confluence, the cells were passaged at the ratio of 1:2. The cells at passage 3 were selected for subsequent adenovirus transduction [22].

### Culture and transfection of MC3T3-E1 cells

Mouse embryo osteoblast precursor cells (MC3T3-E1; American Type Culture Collection, Manassas, VA) were cultured with osteogenic medium (OM). Next, the cells were cultured with Roswell Park Memorial Institute (RPMI)-1640 medium (Gibco Company, Grand Island, NY) containing 10% FBS (Gibco), 10 μg/mL streptomycin, and 100 U/mL penicillin in a 5% CO_2_ incubator (Thermo Fisher Scientific, Waltham, MA) at 37 °C. The cells at the logarithmic growth phrase were digested with trypsin, seeded in 6-well plates at a density of 1 × 10^5^ cells/well, and cultured for 24 h until the confluence reached about 75%.

One day before transfection, the cells were seeded in 6-well plates with 1.5 mL antibiotic-free medium containing 10% FBS. When reaching 30–50% confluence, the cells were transfected. Thereafter, 100 pmol small interfering RNA (siRNA)/2 μg PCNDA3.1-LRP6/HuR was diluted in 250 μL of serum-free DMEM, mixed gently, and allowed to stand at room temperature for 5 min. The sequences of siHuR and NC are 5′-GAGGCAAUUACCAGUUUCAUU-3′, and 5′-UUGUUCGAACGUGUCACGUUU-3′, respectively. Next, 5 μL of transfection reagent Lipofectamine RNAiMAX was diluted in 250 μL of serum-free DMEM, mixed gently, and allowed to stand at room temperature for 5 min. The two dilutions were mixed and left to stand at room temperature for 30 min. The above complex was supplemented to the 6-well plate and cultured for 72 h.

### Establishment of MC3T3-E1 cell line stably transfected with Wnt3a

MC3T3-E1 cells at the logarithmic growth phase were seeded in a 6-well plate (1 × 10^5^ cells/well). Then, 2 μg of the PCDNA3.1-Wnt3a/Vector plasmid was transfected into MC3T3-E1 cells. After 48 h, the cells were screened with 100 μg/mL G418 for one week, with the monoclonal antibodies selected and expanded. The overexpression efficiency of Wnt3a was detected by RT-qPCR and western blot assay. The cell clone with the highest expression efficiency of Wnt3a was used as the MC3T3-E1 cell line stably transfected with Wnt3a for subsequent experiments.

### Cell viability assay

The viability of MC3T3-E1 cells was measured by MTT assay. Cells were seeded in 96-well plates (3.5 × 10^4^ wells). After 24 h of adherence to the wall, the cells were cultured in a renewed medium containing different concentrations of drugs or H_2_O_2_ in a 5% CO_2_ incubator at 37 °C 24 h. MTT (a final concentration of 0.5 mg/mL) was added to incubate with the cells for 4 h in a 5% CO_2_ incubator at 37 °C. Then, 200 μL of dimethyl sulfoxide was added to each well and the OD value was measured with a microplate reader at 570 nm.

### TUNEL assay

Cells were covered with 100 μL equilibration buffer in a 6-well plate and incubated at room temperature for 5–10 min. Cells were then incubated with 50 μL deoxynucleotidyl transferase at 37 °C for 60 min for a plus-tail reaction. Thereafter, the slide was immersed in 2 × SSC and allowed to stand at room temperature for 15 min to stop the reaction. The cells were observed under a fluorescence microscope.

### ALP staining

After 48 h of cell transfection, ALP staining was performed using BCIP/NBT ALP color development kit. Briefly, cells were fixed with 4% paraformaldehyde at 4 °C for 15 min. Next, the cells were stained with the mixture of 3 mL ALP buffer with BCIP solution and nitro blue tetrazolium (NBT) solution at the ratio of 1:2. The cells were added with an appropriate amount of BCIP/NBT staining working solution, and incubated at room temperature in the dark for 5–30 min or longer (up to 24 h) until the development reached the expected degree. The BCIP/NBT staining working solution was removed, and the development was terminated by washing with distilled water.

### P-nitrophenol assay

After 48 h of cell transfection, ALP activity was measured. In short, ALP substrate with a final concentration of 4 mmol/L PNPP was prepared. The cells were lysed with cell lysis buffer containing 50 mM Tris (pH 7.4) and 1% Triton X-100, and sonicated on ice for 5 min. The concentration of protein was determined according to the instructions of a bicinchoninic acid (BCA) kit. The absorbance at 562 nm was measured with a microplate reader, and a standard curve was drawn with protein content (μg) as the abscissa and absorbance as the ordinate. The OD value was detected with a microplate reader at 405 nm, and the protein quantification result was regarded as the relative activity of ALP.

### Alizarin red S staining

Alizarin red S (1 g) was dissolved in 100 mL of ddH_2_O, with the pH adjusted to 4.1–4.3, filtered through a 0.45 μM filter membrane, and stored at room temperature. Cells were fixed with 10% paraformaldehyde for 1 h, rinsed 3 times with ddH_2_O, and incubated with Alizarin red S staining solution for 30 min at 37 °C. The cells were washed 3–5 times with ddH_2_O until the residual staining solution was removed, and then mounted with pre-warmed glycerin gelatin mounting solution at 60 °C. Finally, the cells were photographed with a microscope.

### Cell cycle distribution assay

Cells were trypsinized, fixed overnight with 70% pre-cooled ethanol at − 20 °C, and stored on ice for at least 1 h. The cells were then centrifuged at 4000 rpm for 2 min, resuspended in 0.5 mL of PBS solution containing 0.25% Triton X-100, and incubated for 15 min on ice, followed by another centrifugation at 4000 rpm for 2 min. With the supernatant discarded, the cells were resuspended in 0.5 mL PBS containing 10 μg/mL RNaseA and 20 μg/mL PI solution, and incubated at room temperature in the dark for 30 min. Finally, the cell cycle distribution was detected with flow cytometry.

### Total protein and total RNA extraction from bone tissues

Fresh bone tissue samples were soaked in pre-cooled normal saline and then washed with distilled water to remove blood and red blood cells. The periosteum was removed after which the bone tissues were cut into pieces, weighed, and treated with liquid nitrogen and ground into powder (if it was not convenient to grind using liquid nitrogen, extraction solution could be directly added for grinding). Next, 200–300 μL of protein extraction reagent was added to every 100 mg of bone tissue powder to extract total bone tissue protein. After centrifugation (12,000 rpm) at 4 °C, the collected supernatant was the total protein of bone tissues. The protein was stored in a − 80 °C refrigerator for future use. After the tissue was ground to powder, RNA was extracted using TRIzol reagent. RNA could be used for mRNA isolation, or stored in 70% ethanol at − 70 °C.

### RT-qPCR

Total RNA was extracted from cells or bone tissues using TRIzol reagent (15,596,026, Invitrogen, Carlsbad, CA) and then reverse transcribed into complementary DNA using PrimeScript RT reagent Kit (RR047A, Takara, Japan). RT-qPCR was performed on a PRISM 7300 RT-qPCR System (Applied Biosystems, Foster City, CA) using Fast SYBR Green PCR Kit (Applied Biosystems). GAPDH was used as an internal reference (Additional file 4: Table 1), and the relative expression of genes of interest was analyzed by the 2^−ΔΔCt^ method.

### Western blot assay

Total protein was extracted from cells or bone tissues using enhanced RIPA lysis buffer (Boster Biological Technology, Wuhan, Hubei, China), with the protein concentration determined by the bicinchoninic acid protein quantification kit (Boster). The protein was then separated by 10% sodium dodecyl sulfate–polyacrylamide gel electrophoresis, electro-transferred to a polyvinylidene fluoride membrane, and blocked with 5% BSA at room temperature for 2 h to block non-specific binding. The membrane was probed with diluted primary antibody (Abcam, Cambridge, UK) overnight at 4 °C and then with HRP-labeled secondary antibody at room temperature for 1 min. ECL reagent was used to visualize the results by the X-ray film. ImageJ software was employed to quantify the gray levels of protein bands in western blot images, and β-actin was used as an internal reference.

### Polysome profiling

Sucrose density gradient ultracentrifugation was performed to separate free RNA, 40S ribosomal subunit and 60S ribosomal subunits, assembled 80 s monosome, and mRNA combined with different numbers of ribosomes. After the centrifugation, the high-density Fluorinettm FC-40 reagent was infused from the bottom of the tube. The sucrose solution of different densities, from the lowest at the top layer to the highest at the bottom layer, was obtained using a density gradient separator and transferred to different tubes, followed by detection of optical density at 254 nm. The RNA dissolved in sucrose solution was extracted and purified. The intracellular translation efficiency of LRP6 and GAPDH was represented by the ratio of mRNAs with polyribosome (dissolved in high-concentration sucrose solution) to other mRNAs (dissolved in low-concentration sucrose solution).

### RNA-binding protein immunoprecipitation (RIP) assay

When the cells in a 10 cm dish reached 90% confluence, they were counted, and 2–5 mg protein samples (about 5–20 × 10^8^ cells) was obtained. The antibody was incubated overnight at 4 °C with protein G agarose beads as follows: 20 μL dynabeads protein G, 800 μL NT2 buffer, and 2 μg Anti-HuR. The cells were rinsed with pre-cooled PBS, lysed in lysis buffer with cocktail and RNase I nhibitor on ice for 30 min and centrifuged at 12,000 rpm for 15 min. Next, the supernatant was incubated with antibody-bead complex at 4 °C for 2 h, and added with 1 mL of RIP wash buffer. DNA and protein were digested by DNase-Proteinase K. The cells were incubated with 100 μL NT2 buffer containing DNase I 0.2 μL (1 U/μL) at 37 °C for 15 min, and then with 100 μL NT2 buffer containing 1 μL SDS (10%) and 5 μL proteinase K at 55 °C for 15 min. Finally, the cells were added with 220 μL NT2 buffer, and the supernatant was collected.

### RNA pull-down assay

DNA template was obtained by PCR: The corresponding target fragment was obtained using the PCR method as a template, and a T7 transcription promoter sequence was added to the 5′ end of the forward primer: CCAAGCTTCTAATACGACTCACTATAGGGAGA. Total protein was extracted from cells and incubated in water bath at 37 °C for 2–4 h or with 1 μL DNase I at 37 °C for 30 min. TRIzol reagent was used for purification, with the same procedures as above. Next, 20 μL Dynabeads M-280 magnetic beads were collected, washed twice with 200 μL Solution A (0.1 M NaOH, 0.05 M NaCl) and once with 200 μL Solution B (0.1 M NaOH), then supplemented with 20 μL of TENT buffer (2 × TENT buffer: 20 mM Tris–HCl, pH 8.0, 2 mM EDTA, pH 8.0, 500 mM NaCl, 1% v/v Triton X-100), and put on ice until use.

Reaction working solution was prepared according to the following system: 6 μL RNasin, 500 ng RNA probe, 200 μg cytosolic protein, 2 × TENT buffer, and 75 μL DEPC were hydrated to 150 μL. The sample was incubated for 30 min at room temperature and treated with 20 μL of pre-washed magnetic beads for 30 min at room temperature. After two washes with 200 mL of pre-chilled PBS, the sample was added with 20 μL protein loading buffer, denatured at 95 °C for 5 min, and analyzed by western blot assay.

### RNA half-life analysis

Cells in a 3.5 cm culture dish were transfected with NC and siHuR sequences using the lipofectamine RNAiMAX transfection reagent (5 culture dishes per group). At 3 days after transfection, Act D was added to each culture dish, with a final concentration of 2 μg/mL. Samples were collected at 5 time points: 0 h, 2 h, 4 h, 6 h, and 8 h after dosing. RNA was extracted and analyzed by RT-qPCR.

### Dual-luciferase reporter assay

With plasmids containing the full-length cDNA of the target gene as a template, primers with corresponding endonuclease cleavage sites were designed using Taq DNA polymerase to amplify each target fragment and connected with the corresponding endonuclease cleavage sites of pGL3-promoter vector. The pGL3-promoter recombinant plasmid and the control empty plasmid were co-transfected into HeLa cells. The luciferase activity was measures using the dual-luciferase reporter kit and the GloMax 96-well plate luminescence detector was employed to analyze the results.

### TOPflash (TCF reporter plasmid) assay

MC3T3-E1 cells at the logarithmic growth phase was seeded onto six-well plates (1 × 10^5^ cells/well) and transfected with TOPFlash (D2501-1 μg, Beyotime) and FOPFlash (D2501-100 μg, Beyotime), respectively. After 24-h transfection, the medium was renewed, and the activity of the reporter plasmids were detected using the luciferase reporter detection kit (RG005, Beyotime).

### Telomeric repeat amplification protocol (TRAP) assay and MacNeal’s staining

Bone tissues were routinely hydrated, stained using TRAP staining kit (294–67,001, WAKO, Japan) or MacNeal’s staining kit, and observed under an upright microscope [19].

### Micro-CT detection

The area between the proximal femur and the epiphyseal plate 1–5 mm was scanned using the micro-CT system at a resolution of 16 μm. The area of interest about 29 × 29 × 29 μm^3^ was delineated using a semiautomatic method. The three-dimensional image of the area was reconstructed using the gray values generated by a series of cross-sectional scans. Bone structure parameters in the area, including bone mineral density (BMD), bone volume to total volume (BV/TV), trabecular thickness (Tb.Th.), trabecular number (Tb.N), and trabecular separation (Tb.Sp), were calculated by the system of micro-CT.

### Statistical analysis

SPSS 21.0 statistical software (IBM Corp., Armonk, NY) was used for data processing. Measurement data were presented as mean ± standard deviation. Data obeying normal distribution and homogeneity of variance between two groups were compared using unpaired *t*-test. Comparison among multiple groups was conducted by one-way analysis of variance (ANOVA), followed by Tukey's post hoc tests. Repeated measures ANOVA with Bonferroni post hoc test was applied for the comparison of data at different time points. Difference of *p* < 0.05 was considered to be statistically significant.

## Results

### HuR functioned as a modulator of osteogenic differentiation in OVX-induced mice

**Fig. 1:**
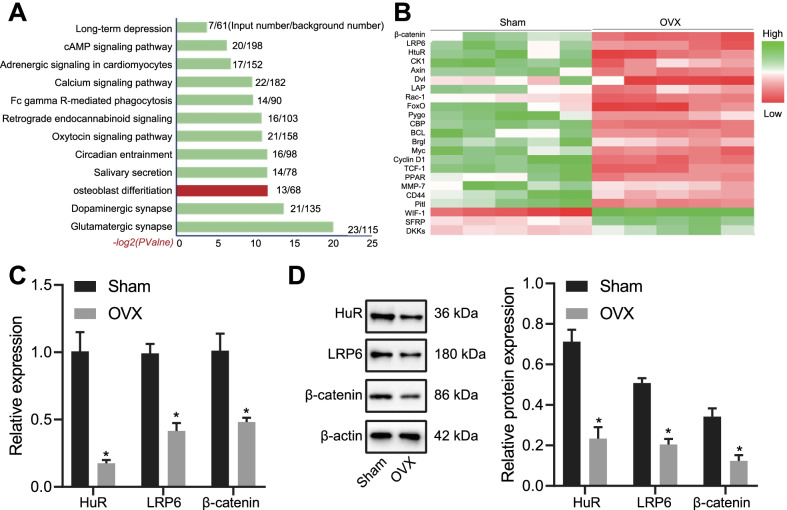
HuR was related to osteogenic differentiation in OVX-induced mice. **a** KEGG enrichment analysis of HuR downstream targets based on bioinformatics analysis. The color of green indicates up-regulated genes, and the color of red indicates down-regulated genes. **b** Expression of osteoporosis-related genes in microarray dataset. **c** RT-qPCR measurement of LRP6, β-catenin, and HuR mRNA expression in sham-operated and OVX-induced mice. D, western blot assay of LRP6, β-catenin, and HuR proteins in sham-operated and OVX-induced mice. *p < 0.05 versus sham-operated mice. Measurement data were expressed as mean ± standard deviation. Data between two groups were compared using unpaired *t*-test

First, potential HuR targets were identified by taking the intersection of HuR downstream targets searched from the CLIPdb2 database, putative target mRNAs obtained using Piranha and CIMS tools, and prediction results of the RPISeq website. The identified candidates were then subjected to KEGG pathway enrichment analysis, through which HuR was found to be correlated with osteogenic differentiation (Fig. 1a). In addition, the results of bioinformatics analysis presented that the expression of bone formation-related genes including β-catenin and LRP6 were down-regulated and expression of HuR was also reduced in OVX mice (Fig. 1b).

Furthermore, results of RT-qPCR and western blot analyses confirmed that the mRNA and protein expression of LRP6, β-catenin, and HuR was decreased in the bone tissues of OVX mice (Fig. 1c, d).

These results demonstrated the diminished expression of HuR along with the decreases in levels of osteogenesis-related LRP6 and β-catenin in the bone tissues of mice after ovariectomy, which suggests that HuR might be an upstream regulatory protein affecting osteogenic differentiation.

### Silencing of HuR inhibited the differentiation of MC3T3-E1 cells into osteoblasts

**Fig. 2:**
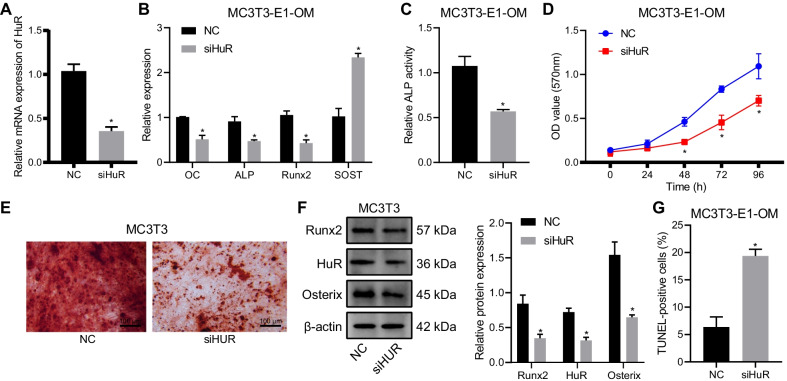
HuR silencing prevented osteogenic differentiation of MC3T3-E1 cells while promoting cell apoptosis. **a** RT-qPCR detection of the silencing efficiency of siHuR treatment. **b** RT-qPCR detection of osteoblast-related markers in MC3T3-E1 cells with HuR silencing. **c** Detection of ALP activity by p-nitrophenol assay in MC3T3-E1 cells with HuR silencing. **d** Detection of cell viability by MTT assay in MC3T3-E1 cells with HuR silencing. **e** Detection of the number of calcium nodules in MC3T3-E1 cells with HuR silencing by Alizarin red S staining. **f** Western blot determination of of osteogenic differentiation-related proteins (Runx2 and Osterix) in MC3T3-E1 cells with HuR silencing. **g** Detection of cell apoptosis in MC3T3-E1 cells with HuR silencing by TUNEL staining. OM, osteogenic medium. *p < 0.05 versus the NC group. Measurement data were expressed as mean ± standard deviation. Data between two groups were compared using unpaired *t*-test. Statistical analysis in relation to time-based measurements within each group was realized using repeated measures ANOVA, followed by a Bonferroni’s post hoc test. The cellular experiment was repeated three times independently

To study the effect of HuR on the differentiation ability of MC3T3-E1 cells, the cells were cultured with OM and transfected with siHuR, followed by detection of the osteoblast marker expression. The down-regulated HuR expression following siHuR treatment was confirmed by RT-qPCR (Fig. 2a). RT-qPCR results then displayed that the expression of osteocalcin (OC), ALP, and Runx2 was repressed in siHuR-treated MC3T3-E1 cells, while that of sclerostin (negative regulator of bone formation) was increased (Fig. 2b), indicating that the osteogenesis was repressed in the presence of knockdown of HuR.

Meanwhile, the p-nitrophenol assay data exhibited that the activity of ALP, a marker of mature osteoblasts, was attenuated upon knockdown of HuR (Fig. 2c), indicating the suppression of osteogenic differentiation due to the silencing of HuR. Moreover, MTT assay data illustrated the cell viability was decreased upon HuR silencing (Fig. 2d). Additionally, Alizarin red S staining revealed that the number of calcium nodules was reduced in MC3T3-E1 cells following knockdown of HuR (Fig. 2e). In addition, the protein expression of Runx2 and Osterix was diminished in MC3T3-E1 cells in response to HuR knockdown (Fig. 2f). Cell apoptosis, as assessed by TUNEL staining, was augmented in the presence of HuR silencing (Fig. 2g).

Taken together, knocking down HuR could inhibit differentiation of MC3T3-E1 cells into osteoblasts and accelerate MC3T3-E1 cell apoptosis.

### HuR bound to the 3’UTR of LRP6 mRNA

**Fig. 3:**
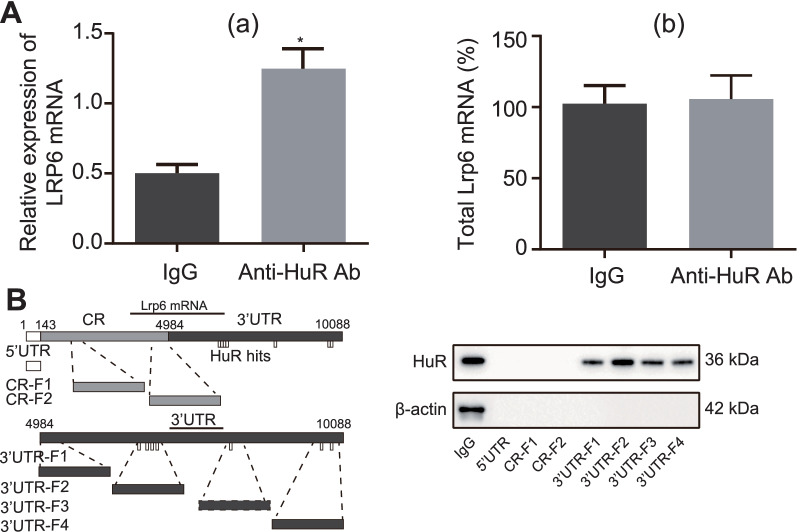
HuR bound to the 3'UTR of LRP6 mRNA. **A** Detection of binding affinity of HuR to LRP6 mRNA by RIP assay. **B** Binding affinity of biotin-labeled LRP6 mRNA to HuR in different segments detected by RNA pull-down assay. * *p* < 0.05 versus the IgG group. Measurement data were expressed as mean ± standard deviation. Data between two groups were compared using unpaired *t*-test. The cellular experiment was repeated three times independently

After observing the effect of HuR on MC3T3-E1 cell differentiation and apoptosis, we then aimed to investigate the underlying downstream mechanism. RIP data revealed that LRP6 mRNA was significantly enriched in cells incubated with anti-HuR antibody (approximately 10 folds) (Fig. 3Aa); in contrast, the level of LRP6 mRNA was similar in directly extracted Input RNA from cell lysate treated with anti-IgG antibody and anti-HuR antibody without antibody incubation (Fig. 3Ab).

Further to determine the binding region of HuR to the LRP6 mRNA, LRP6 mRNA was segmented, including 5'UTR, coding region (CR), 3'UTR-F1, 3'UTR-F2, 3'UTR-F3, and 3'UTR-F4, labeled with biotin and incubated with cell lysate. Magnetic beads with avidin were used to enrich relevant fragments and their binding proteins. The results of western blot assay identified the binding between HuR and the 3'UTR-F2 fragment of LRP6 mRNA (Fig. 3B).

Taken together, HuR could bind to the 3’UTR of LRP6 mRNA.

### HuR stabilized LRP6 mRNA and promoted its translation

**Fig. 4:**
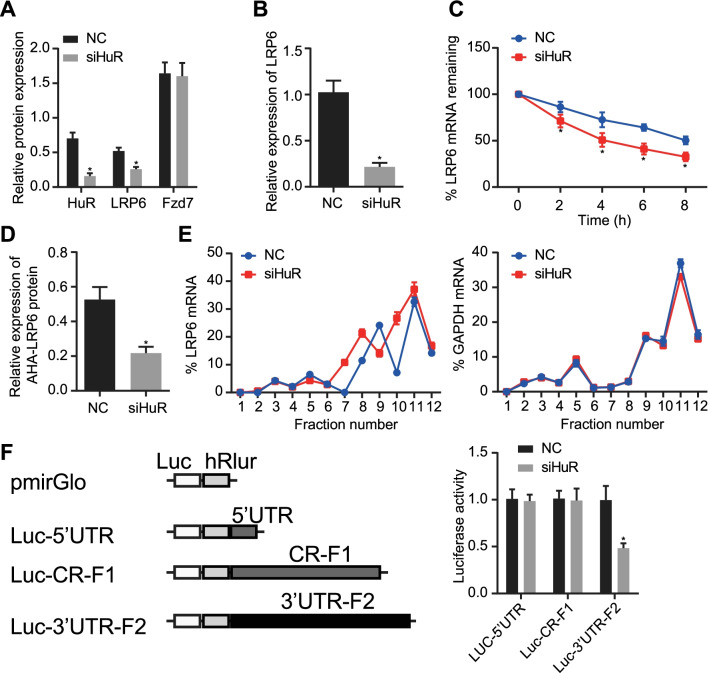
HuR stabilized LRP6 mRNA and enhanced its translation. **a** Detection of LRP6 expression in MC3T3-E1 cells with HuR silencing by western blot assay. **b** Detection of LRP6 mRNA expression in MC3T3-E1 cells with HuR silencing by RT-qPCR. **c** Detection of LRP6 mRNA stability in response to HuR knockdown detected by half-life assay. **d** In vitro translation detection of LRP6 protein synthesis after HuR knockdown. E, Detection of LRP6 mRNA fragments by ribosome translation. **f** Binding of HuR to 3’-UTR of LRP6 mRNA as validated by dual-luciferase reporter assay. * *p* < 0.05 versus the NC group. Measurement data were expressed as mean ± standard deviation. Data between two groups were compared using unpaired *t*-test. The cellular experiment was repeated three times independently

Knockdown of HuR in MC3T3-E1 cells repressed the protein and mRNA expression of LRP6 without affecting that of its binding protein Frizzled-7 (Fzd7) (Fig. 4a, b). To study the effect of HuR on the stability of LRP6 mRNA, MC3T3-E1 cells were treated with actinomycin D (ActD) after transfection of siHuR to inhibit RNA synthesis, followed by detection of the half-life of LRP6 mRNA. The results showed that HuR knockdown resulted in a decrease in the half-life of LRP6 mRNA (Fig. 4c). Meanwhile, silencing of HuR repressed the synthesis of LRP6 protein (Fig. 4d). Subsequently, a polysomal profile was performed to detect the translation status of LRP6 mRNA. It was found that HuR knockdown led to decreased translation of LRP6 mRNA components (components 6–8) (Fig. 4e). The results of dual-luciferase reporter assay then substantiated that HuR bound to the 3’UTR-F2 segment of LRP6 mRNA (Fig. 4f). In summary, HuR could stabilize LRP6 mRNA and promote its translation.

### HuR enhanced the osteogenic differentiation while reducing the apoptosis of MC3T3-E1 cells by regulating LRP6

**Fig. 5:**
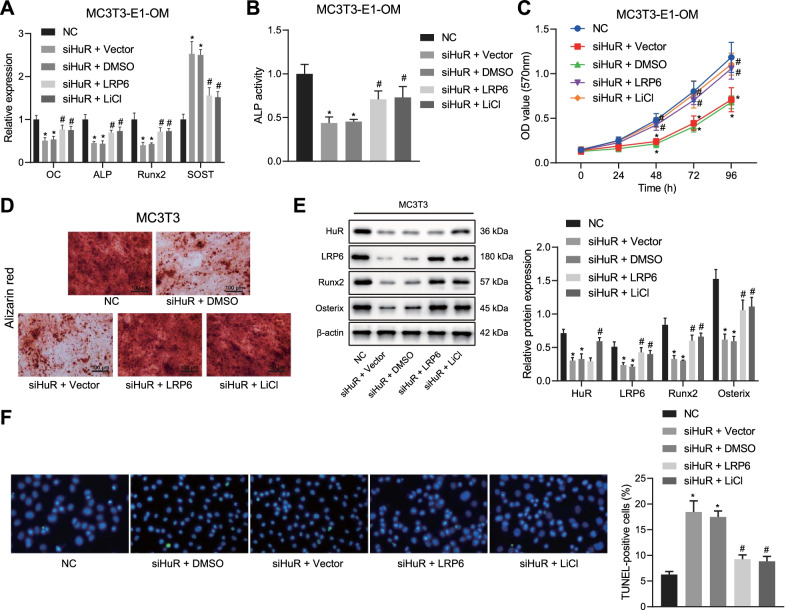
HuR enhanced MC3T3-E1 cell osteogenic differentiation and arrested its apoptosis via modulating LRP6. **a** RT-qPCR measurement of osteoblast-related marker expression in MC3T3-E1 cells in response to siHuR alone or in combination with LRP6 overexpression or LiCl. **b** Detection of ALP activity in MC3T3-E1 cells by p-nitrophenol assay in response to siHuR alone or in combination with LRP6 overexpression or LiCl. **c** Detection of MC3T3-E1 cell viability by MTT assay in response to siHuR alone or in combination with LRP6 overexpression or LiCl. **d** Detection of the number of calcium nodules in MC3T3-E1 cells by Alizarin red S staining in response to siHuR alone or in combination with LRP6 overexpression or LiCl. **e** Western blot measurement of HuR, LRP6 and osteogenesis-related proteins (Runx2 and Osterix) in response to siHuR alone or in combination with LRP6 overexpression or LiCl. **f** Detection of apoptosis of MC3T3-E1 cells by TUNEL staining in response to siHuR alone or in combination with LRP6 overexpression or LiCl. *p < 0.05 versus the NC group, ^#^*p* < 0.05 versus the siHuR + Vector or siHuR + DMSO groups. Measurement data were expressed as mean ± standard deviation. Data comparison among multiple groups was conducted by one-way ANOVA, followed by Tukey’s post hoc test. The cellular experiment was repeated three times independently

Next, we sought to probe whether HuR regulates osteogenic differentiation through regulation of LRP6. After transfection of siHuR in MC3T3-E1 cells, the expression of OC, ALP, and Runx2 mRNA was diminished, whereas the expression of sclerostin was augmented. These effects of HuR knockdown alone were reversed in response to additional overexpression of LRP6 or treatment of LiCl, an agonist of the Wnt pathway (Fig. 5a).

Moreover, ALP activity was reduced in response to siHuR, which was negated following additional overexpression of LRP6 or treatment with LiCl (Fig. 5b). The attenuating effect of HuR knockdown alone on cell viability was also abolished by overexpression of additional LRP6 or treatment of LiCl (Fig. 5c).

As shown in Fig. 5d, Alizarin red S staining assay displayed that the number of calcium nodules was reduced in MC3T3-E1 cells silencing HuR, and such reduction was reversed in the presence of additional overexpression of LRP6 or treatment of LiCl. In addition, western blot assay uncovered that knockdown of HuR diminished the expression of Runx2 and Osterix, which could be counteracted when LRP6 was overexpressed or LiCl was added (Fig. 5e). Furthermore, the promoting effect of siHuR treatment alone on cell apoptosis was reversed upon its combination with LRP6 overexpression or LiCl (Fig. 5f).

To sum up, LRP6 overexpression reversed the inhibitory effect of HuR silencing on the osteogenic differentiation of MC3T3-E1 cells and the promoting effect on the cell apoptosis, which indicated that HuR could enhance the osteogenic differentiation while attenuating the apoptosis of MC3T3-E1 cells by regulating LRP6.

### HuR or LRP6 knockdown decreased cell viability of Wnt3a-overexpressing MC3T3-E1 cells

**Fig. 6:**
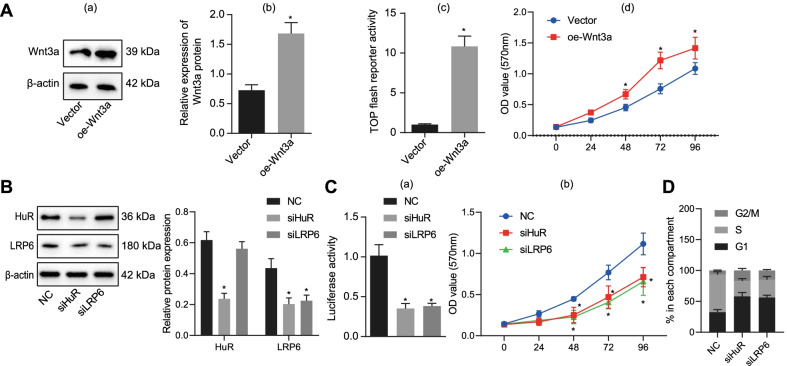
Knockdown of HuR or LRP6 repressed cell viability in MC3T3-E1 cells overexpressing Wnt3a. **A** Representative experimental image (a) and the corresponding quantitative analysis (b) in western blot measurement of Wnt3a expression, the activation of the Wnt pathway detected with TOPFLASH reporter gene assay (c) and the cell viability in MC3T3-E1 cells stably transfected with oe-Wnt3a as detected with MTT assay (d). **B** Detection of HuR and LRP6 expression by western blot assay in MC3T3-E1 cells transfected with siHuR or siLRP6. **C** Detection of Wnt pathway activation and cell viability in MC3T3-E1 cells by dual-luciferase reporter assay and MTT assay. **D** Flow cytometric analysis of cell cycle distribution in MC3T3-E1 cells. *p < 0.05 versus the Vector or NC groups. Measurement data were expressed as mean ± standard deviation. Data between two groups were compared using unpaired *t*-test. Data comparison among multiple groups was conducted by one-way ANOVA, followed by Tukey’s post hoc test. The cellular experiment was repeated three times independently

We further moved to investigate whether the regulation of HuR/LRP6 affected the Wnt pathway and the cell viability, of Wnt3a-overexpressing MC3T3-E1 cells. The protein expression of Wnt3a was successfully increased in the cells treated with oe-Wnt3a (Fig. 6Aa–b), leading to enhanced activation of the Wnt pathway (Fig. 6Ac) and augmented cell viability (Fig. 6Ad).

In addition, MC3T3-E1 cells silencing HuR or LRP6 were established through siHuR or siLRP6 treatment (Fig. 6B). The knockdown of either HuR or LRP6 resulted in weakened activation of the Wnt pathway (Fig. 6Ca), along with reduced cell viability (Fig. 6Cb). Flow cytometric analysis identified that the proportion of cells arrested at the S phase was diminished after HuR knockdown or LRP6 knockdown, whereas that of cells arrested at the G2/M and G1 phases was increased (Fig. 6D).

To sum up, silencing of HuR or LRP6 could attenuate MC3T3-E1 cell viability by blocking the activation of the Wnt pathway.

### Overexpression of HuR alleviated osteoporosis-related phenotypes in OVX mice

**Fig. 7:**
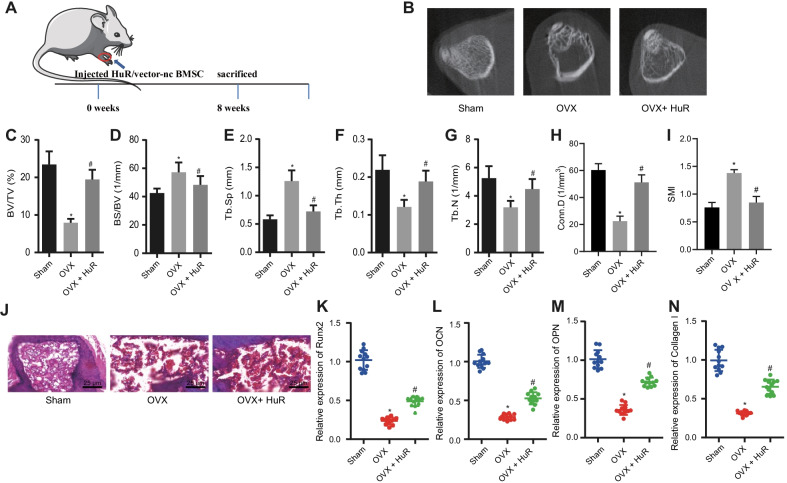
Overexpression of HuR ameliorated osteoporosis-related phenotypes in OVX mice. **a** A graph illustrating animal model and treatment. **b** Morphology of femur tissues of sham-operated mice, untreated OVX mice, or OVX mice treated with oe-HuR scanned by micro-CT. C-I, Quantitative analysis of BV/TV (**c**), BS/BV (**d**), Tb.Sp (**e**), Tb.Th (**f**), Tb.N (**g**), Conn.D (**h**), and structural model index (SMI) (**i**) in sham-operated mice, untreated OVX mice, or OVX mice treated with oe-HuR by micro-CT scan. **j** HE staining to observe the osteogenic differentiation in sham-operated mice, untreated OVX mice, or OVX mice treated with oe-HuR. **k–n** Detection of mRNA expression of osteogenic differentiation-related factors Runx2 (**k**), OCN (**l**), OPN (**m**), COL1 (**n**) in bone tissues of sham-operated mice, untreated OVX mice, or OVX mice treated with oe-HuR by RT-qPCR. *p < 0.05 versus sham-operated mice, # *p* < 0.05 *vs.* Untreated OVX mice. Measurement data were expressed as mean ± standard deviation. Data comparison among multiple groups was conducted by one-way ANOVA, followed by Tukey’s post hoc test. n = 12

The aforementioned results suggested that after ovariectomy, the expression of HuR was diminished in bone tissues of mice. To investigate whether overexpression of HuR could alleviate the phenotypes of OVX-induced osteoporosis in vivo, we isolated primary BMSCs from mice, established HuR overexpressing BMSCs and injected the cells into the mice (Fig. 7a).

The expression of both LRP6 and HuR was significantly reduced in the bone tissues of the OVX mice when compared with the sham-operated mice. Additional treatment of oe-HuR increased the LRP6 and HuR expression (Additional file 1: Fig. 1A–D). Three weeks later, micro-CT was employed to observe femoral tissue-related phenotypes. The Tb.N in OVX mice was reduced while it was partially restored after overexpression of HuR (Fig. 7b). Further, overexpression of HuR was found to alleviate the decrease in the BV/TV, Tb.Th, Tb.N, and trabecular connectivity density (Conn.D) in OVX mice as well as the increase in the BS/BV, Tb.Sp and SMI (Fig. 7c–i). These results indicated that HuR overexpression led to an increase in OVX mice.

Meanwhile, HE staining results showed that overexpression of HuR could reduce bone gap and accelerate bone proliferation in OVX mice (Fig. 7j). Additionally, analysis on the bone tissues stained with TRAP uncovered that HuR overexpression decreased the number of osteoclasts as well as TRAP-positive cells in the metaphysis of the distal femur (Additional file 2: Fig. 2A). Alizarin red S staining data revealed that the differentiation of osteoblasts in the bone tissues of the OVX mice was decreased, and this decrease was reversed by HuR overexpression (Additional file 2: Fig. 2B). These results indicated that HuR overexpression could trigger osteoblast formation.

Furthermore, the results of RT-qPCR revealed that HuR overexpression abolished the decrease of Runx2, OCN, OPN, and COL1 mRNA expression induced by OVX (Fig. 7k–n). Consistent results were obtained in ELISA assay (Additional file 3: Fig. 3).

Taken together, HuR could alleviate osteoporosis-related phenotypes in OVX mice, including reduced bone mass, osteoblast mineralization ability, and collagen synthesis ability.

## Discussion

**Fig. 8:**
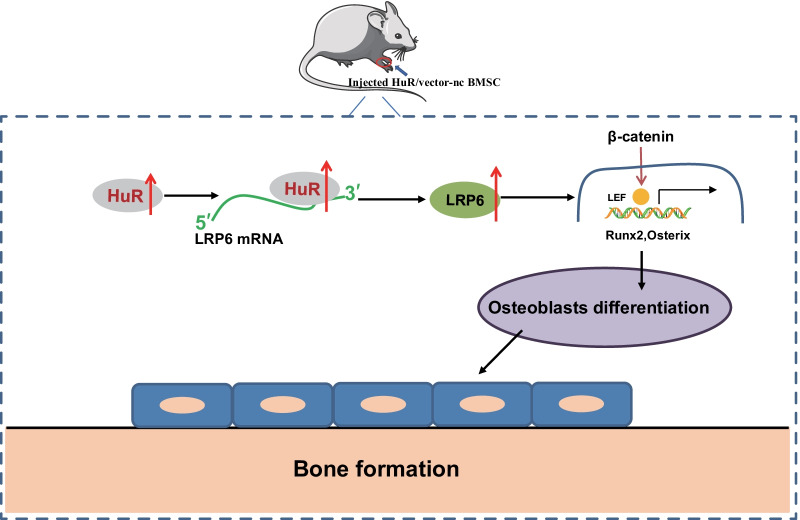
Mechanism graph of the regulatory network and function of HuR in bone formation. Overexpression of HuR elevates the expression of LRP6 by activating the Wnt pathway, facilitating osteogenic differentiation and bone formation, and thus arresting osteoporosis

Both in vitro and in vivo findings in the current study suggested that RNA-binding protein HuR could potentially promote osteogenic differentiation and thus alleviated osteoporosis progression by promoting LRP6 expression at protein level (Fig. 8).

The obtained findings unveiled that HuR expression was decreased in the bone tissues of OVX-induced mice and its silencing prevented the differentiation of MC3T3-E1 cells into osteoblasts. Runx2 and Osterix are essential for osteogenic differentiation and their increased expression is associated with the enhancement of osteogenic differentiation [23, 24]. In addition to Runx2 and Osterix, ALP activity has been also a well-established marker of osteogenic differentiation whereby its inhibition indicates the suppression of osteogenic differentiation of MC3T3-E1 cells [25, 26]. Here, the current results showed that silencing of HuR could result in decreased expression of Runx2 and Osterix along with reduced ALP activity in MC3T3-E1 cells. Combining the published reports with the obtained results from this study, we are convinced that HuR could act as a promoter to facilitate osteogenic differentiation. Meanwhile, in vivo data in this investigation suggested that HuR overexpression promoted bone formation due to its alleviating role in the decrease in the expression of bone formation-related genes (Runx2, OCN, OPN, and COL1) induced by OVX. Indeed, OCN, OPN, and COL1 are osteogenic phenotypic markers, the upregulation of which is associated with the promotion of bone formation [27, 28]. Meanwhile, HuR overexpression could disrupt the osteoporosis-related phenotypes, including reduced bone mass, osteoblast mineralization, and collagen synthesis. Therefore, HuR may have the potential to serve as a therapeutic target against osteoporosis.

Additionally, the present study confirmed that the therapeutic effect of HuR on the osteoporosis was achieved by the upregulation of LRP6. Specifically, HuR can bind to the 3’UTR of LRP6 mRNA, and consequently promoted the stability of LRP6 mRNA and its translation, thus inducing osteogenic differentiation and arresting the development of osteoporosis. Published literature has reported that HuR functions as a post-transcriptional regulator of gene expression mainly due to its RNA-binding activity [29]. In addition, at the molecular level, HuR can bind to the LRP6 mRNA via its 3’UTR and increases LRP6 expression by stabilizing LRP6 mRNA and stimulating its translation; meanwhile, HuR deficiency can induce a decrease in the expression of LRP6 in the mucosal tissues [9]. Additionally, LRP6 has been shown to promote the osteogenic differentiation of osteoblasts [30]. LRP6 has been also essential for normal postnatal osteogenesis and osteoblast function [31]. A previous study has elucidated that single nucleotide polymorphisms of the LRP6 gene are associated with the femoral neck BMD [32]. Taken together, this study represents the first evidence for the post-transcriptional regulation of LRP6 by HuR in osteoblasts and may have importance in regulating osteoporosis progression.

Further analysis revealed that HuR promoted the expression of LRP6 to activate the Wnt pathway, thereby augmenting the osteogenic differentiation. LRP6 participates in an array of biological processes as an important Wnt ligand receptor that regulates β-catenin-related signaling [33]. Additionally, it has been reported that LRP6 is an important co-receptor Wnt/β-catenin upstream pathway [34, 35]. Currently, two main bone anabolic pathways have been identified: canonical WNT (cWNT) signaling and parathyroid hormone (PTH) signaling [36]. A recent study has substantiated that the Wnt pathway agonist SKL2001 can promote the osteogenic differentiation of rat ectomesenchymal stem cells [37]. In addition, the Wnt pathway has demonstrated an important role in promoting the osteogenic differentiation of osteoblasts under oxidative conditions [38]. These data provide evidence that targeting the HuR/LRP6/Wnt axis may aid in the development of more effective therapeutic strategies to prevent osteoporosis. In relation to our findings, a specific regulatory network has been established by which the binding of the Wnt proteins to the Fzd-LRP5/6 co-receptor complex could recruit disheveled (Dvl), a cytoplasmic signaling protein, to the cytoplasmic tail of Fzd; and Dvl could in turn lead to LRP5/6 cytoplasmic tail phosphorylation as well as the inhibition of the β-catenin destruction complex [39]. Whether this mechanism interacts with the HuR/LRP6/Wnt axis delineated in our study merits further investigation. Meanwhile, due to the lack of supporting literature elucidating the interaction between HuR and Wnt pathway, further studies are needed to validate the findings of our study.

## Conclusion

In conclusion, HuR can promote the expression of LRP6 and thereby activate the downstream Wnt pathway, facilitating osteogenic differentiation, thus relieving osteoporosis progression. This study helps to deepen our understanding of the pathogenesis of osteoporosis. However, the current study only presents preclinical evidence of the role played by HuR in bone formation and its potential as a drug target. Further clinical trials are needed for the development of HuR-based therapeutic agents.

## Supplementary Information

**Additional file 1: Fig. 1**. HuR overexpression up-regulated LRP6 expression in OVX mice. RT-qPCR (A, C) and western blot (B, D) analyses of the expression of LRP6 (A, B) and HuR (C, D) in bone tissues of sham-operated mice, untreated OVX mice, or OVX mice treated with oe-HuR. * p < 0.05 vs. sham-operated mice, # p < 0.05 vs. Untreated OVX mice. Measurement data were expressed as mean ± standard deviation. Data comparison among multiple groups was conducted using one-way ANOVA with Tukey's post hoc test. n = 12.

**Additional file 2: Fig. 2**. Analysis of the number of osteoclasts and osteoblasts in the presence of HuR overexpression in OVX mice. A, TRAP staining analysis of the number of osteoclasts in bone tissues of sham-operated mice, untreated OVX mice, or OVX mice treated with oe-HuR (TRAP-positive cells are located at the distal femoral metaphysis, as indicated by arrows); B, Alizarin red S staining analysis of the differentiation of osteoblasts in bone tissues of sham-operated mice, untreated OVX mice, or OVX mice treated with oe-HuR. * p < 0.05 vs. sham-operated mice, # p < 0.05 vs. OVX mice. Measurement data were expressed as mean ± standard deviation. Data comparison among multiple groups was conducted using one-way ANOVA with Tukey's post hoc test. n = 12.

**Additional file 3: Fig. 3**. ELISA measurement of the production of osteoblast- and the Wnt pathway-related factors in the serum of sham-operated mice, untreated OVX mice, or OVX mice treated with oe-HuR. * p < 0.05 vs. sham-operated mice, # p < 0.05 vs. OVX mice. Measurement data were characterized as mean ± standard deviation. Data comparison among multiple groups was conducted using one-way ANOVA with Tukey's post hoc test. n = 12.

**Additional file 4: Supplementary Table 1**. Primer sequences for RT-qPCR.

## Data Availability

The datasets generated/analyzed during the current study are available.

## Abbreviations

HuR: Human antigen R
LRP6: Lipoprotein receptor-related protein 6
BMSCs: Bone marrow-derived mesenchymal stem cells
OVX: Ovariectomized
CPE: Cytopathic effect
P1: Passage 1
H&E: Hematoxylin and eosin
ELISA: Enzyme-linked immunosorbent assay
DMEM: Dulbecco's modified Eagle's medium
P0: Passage 0
OM: Osteogenic medium
RPMI: Roswell Park Memorial Institute
siRNA: Small interfering RNA
NBT: Nitro blue tetrazolium
BCA: Bicinchoninic acid
RIP: RNA-binding protein immunoprecipitation
TRAP: Telomeric repeat amplification protocol
BMD: Bone mineral density
BV/TV: Bone volume to total volume
Tb.Th.: Trabecular thickness
Tb.N: Trabecular number
Tb.Sp: Trabecular separation
ANOVA: Analysis of variance
OC: Osteocalcin
CR: Coding region
ActD: Actinomycin D
Conn.D: Connectivity density
cWNT: Canonical WNT
PTH: Parathyroid hormone
Dvl: Disheveled
