# Supplementary material for: Potential of RNA-binding protein human antigen R as a driver of osteogenic differentiation in osteoporosis
Source: J Orthop Surg Res. 2022 Apr 12;17:234. doi: 10.1186/s13018-022-03073-w (PMC9003960; doi:10.1186/s13018-022-03073-w)
Supplement: Supplementary file 4 — Additional file 4: Supplementary Table 1. Primer sequences for RT-qPCR. [file 13018_2022_3073_MOESM4_ESM.docx]

**Supplementary Table 1** Primer sequences for RT-qPCR

| Gene | Primer sequence |
| --- | --- |
| HuR | Forward: 5’-GGATGACATTGGGAGAACGAAT-3’ |
|  | Reverse: 5’-TGTCCTGCTACTTTATCCCGAAT-3’ |
| ALP | Forward: 5’-CCAACTCTTTTGTGCCAGAGA-3’ |
|  | Reverse: 5’-GGCTACATTGGTGTTGAGCTTTT-3’ |
| LRP6 | Forward: 5’-TGCAAACAGACGGGACTTGAG-3’ |
|  | Reverse: 5’-CGGGGACAATAATCCAGAAACAA-3’ |
| Osterix | Forward: 5’-GGAAAGGAGGCACAAAGAAGC-3’ |
|  | Reverse: 5’-CCCCTTAGGCACTAGGAGC-3’ |
| OCN | Forward: 5’-CTGACCTCACAGATCCCAAGC-3’ |
|  | Reverse: 5’-TGGTCTGATAGCTCGTCACAAG-3’ |
| OPN | Forward: 5’-ATCTCACCATTCGGATGAGTCT-3’ |
|  | Reverse: 5’-TGTAGGGACGATTGGAGTGAAA-3’ |
| COL1 | Forward: 5’-GCTCCTCTTAGGGGCCACT-3’ |
|  | Reverse: 5’-ATTGGGGACCCTTAGGCCAT-3’ |
| GAPDH | Forward: 5’-AGGTCGGTGTGAACGGATTTG-3’ |
|  | Reverse: 5’-GGGGTCGTTGATGGCAACA-3’ |

Note: HuR, human antigen R; ALP, alkaline phosphatase; LRP6, lipoprotein receptor-related protein 6; OCN, osteocalcin; OPN, osteopontin; GAPDH, glyceraldehyde-3-phosphate dehydrogenase; RT-qPCR, reverse transcription quantitative polymerase chain reaction
